# Supplementary material for: A reverse vaccinology approach identifies putative vaccination targets in the zoonotic nematode Ascaris
Source: Front Vet Sci. 2022 Nov 1;9:1014198. doi: 10.3389/fvets.2022.1014198 (PMC9665164; doi:10.3389/fvets.2022.1014198)
Supplement: Supplementary Table 2 — Protein sequences predicted to have epitopes that bind to all 27 MHC-II alleles, used as a reference set, with a score between 0.01 and 1 in the MHCII-IEDB tool. [file Table_2.pdf]

Supplementary Table 2 - Protein sequences predicted to have epitopes that bind to all 27 MHC-II alleles, used as a reference set, with a score between 0.01 and 1 in the MHCII-IEDB tool.

**Species**	**Bioproject**	**WormBase Protein transcript identifier**	**Protein transcript length (aa)**	**Phobius sub-cellular location**
A. lumbricoides	PRJEB4950	ALUE_0000401101	3420	Transmembrane
		ALUE_0000418301	2196	Transmembrane
		ALUE_0000418601	2591	Transmembrane
		ALUE_0000827601	3725	Transmembrane
		ALUE_0000834301	3758	Transmembrane
		ALUE_0000985601	2181	Transmembrane
		ALUE_0001005801	1141	Transmembrane
		ALUE_0001162401	2044	Transmembrane
		ALUE_0001794801	774	Transmembrane
A. suum	PRJNA80881	GS_03113	2294	Transmembrane
		GS_05892	3963	Transmembrane
		GS_21459	1022	Transmembrane
	PRJNA62057	AgB13X_g094_t03	2057	Transmembrane
		AgB13X_g094_t04	2060	Transmembrane
		AgB13X_g094_t05	1862	Transmembrane
		AgB13X_g096_t02	2637	Transmembrane
		AgR002_g353_t01	2173	Transmembrane
		AgR007_g063_t01	2612	Transmembrane
		AgR007_g063_t02	2661	Transmembrane
		AgR007_g063_t03	2670	Transmembrane

	AgR007_g063_t04	2682	Transmembrane
	AgR007_g063_t06	2666	Transmembrane
	AgR007_g282_t01	1891	Transmembrane
	AgR007_g282_t02	1889	Transmembrane
	AgR007_g282_t14	1770	Transmembrane
	AgR028_g099_t06	2170	Secreted
	AgR028_g099_t07	2631	Secreted
	AgR035X_g027_t05	3668	Transmembrane
	AgR035X_g062_t03	3452	Secreted
	AgR052_g054_t02	1386	Transmembrane
	AgR052_g054_t03	1412	Transmembrane
	AgR052_g054_t04	1384	Transmembrane
