# Supplementary material for: A reverse vaccinology approach identifies putative vaccination targets in the zoonotic nematode Ascaris
Source: Front Vet Sci. 2022 Nov 1;9:1014198. doi: 10.3389/fvets.2022.1014198 (PMC9665164; doi:10.3389/fvets.2022.1014198)

S4. **A.** Location of the selected Th cell and B cell epitopes using the best expressed transcripts for each selected antigen. The location for each glycosylation site in each transcript, as predicted by NetNGlyc 1.0, is also provided.

APiezo - AgR007_g063_t01

Th cell Epitope location: 926-940 and 1157-1171

B cell epitope location: 2453-2468 and 2490-2504

NetNGlyc 1.0 – 674, 764, 841, 853, 914, 1069, 2353

AProto - AgB13X_g096_t06

Th cell Epitope location: 588-602 and 754-768

B cell epitope location: 1294-1310 and 1747-1781

NetNGlyc 1.0 – 185, 228, 393, 428, 510, 671, 787, 951, 1086, 1159, 1253, 1424, 1445, 1621, 1645, 1676, 1842, 1872, 2004, 2229

ALtype - AgR007_g282_t05

Th cell Epitope location: 65-79 and 1285-1299

B cell epitope location: 1029-1040 and 1275-1282

NetNGlyc 1.0 – 332, 800, 1449

ATtype - AgB13X_g094_t05

Th cell epitope location: 1344-1358 and 1742-1756

B cell epitope location: 1262-1270 and 1462-1475

NetNGlyc 1.0 – 248, 326, 372, 383, 549, 55, 585, 615, 836, 1412, 1661, 1844

S4 B. Raw output of NetNGlyc 1.0 for the highest transcribed transcripts for each selected antigen.

NetNGlyc-1.0 Server Output - DTU Health Tech

Asn-Xaa-Ser/Thr sequons in the sequence output below are highlighted in **blue**.

Asparagines predicted to be N-glycosylated are highlighted in **red**.

Output for 'AgR007_g063_t01'

```
##############################################################################
#############

    Warning: This sequence may not contain a signal peptide!!

    Proteins without signal peptides are unlikely to be exposed to
    the N-glycosylation machinery and thus may not be glycosylated
    (in vivo) even though they contain potential motifs.

    SignalP-NN euk predictions are as follows:

  # name                  Cmax  pos ?  Ymax  pos ?  Smax  pos ?  Smean ?  D
?
  AgR007_g063_t01              0.186  24  0.197  11  0.446   1  0.348   0.258
N  0.500      SignalP-TM

    SignalP output is explained at
https://services.healthtech.dtu.dk/services/SignalP-4.1/output.php

##############################################################################
#############

Name:  AgR007_g063_t01         Length:  2612
MPFDQRAYFLLYYALLPIVLVTACTLRINIFSFFYGLFLLFLPFSLSSLNSDLGHPKKFLLCLTLSSFVFTLAQI
AFNAV      80
TWIDVPEENEFHGCVAVEDRLREFGLERLSLNNLVDTMGSLAPNVIVFITASLLVIRILLTEHQKDRNLLTVELE
KIRES      160
DGQLQSQILRIISNAKWSSLMTQLHDVLFCIMLAIAGAIHPSLLSAVYFLISVVIVICWAFDYTPTSAQFRTLRQ
YVLVL      240
SGFHVALIYLYQMPTMRTFCEPLSTIARLFGLVYLKPTKCDPQPSIRVTPYWPWSQMLSPYAYLLLYSLVAIQLR
VVQYS      320
GLQKSDYAEEQSLYLAQSQLRGLTNEKSFPSVTKRNLMEQTYNMARSQSYVLMLFVMMAWSLLYHSWLTFVLMIV
SCVVW      400
MYPNSQRFCLKISPYIAVYAQALVLIQFVYGLDLTQAELPDEKPVFLRQIGLEKSRLSPPWRPILLKFTFTVLFW
VTVKQ      480
RTKERRRGDDEEIETPEKAEIDAESPLKGEQPLQPSLTAINPAVSYLRHLVTKYWVVVNLCLLLAISLQNPVVFY
RIIYM      560
AFFQIFVNCFQISFPTWRKSLYAFWTFMVAYCMFVLSLIYTYQFHGFPQLYQQYLGMSEDVLRSFGLERFSSGQL
FIRLL      640
```

TPISFLIFTLVQMNFFHENLMERTGKWEKAFYHNQSRLSTMLGKFHKRHGGLLNIVKVAISPSATSAKDEEILDG
KVDSS 720

PKASVKIRIEEPEEERNFPRVKEYIDIIGERCRSFADYHELIVNYTWRFIEIHIDKAIAFMMLHICVSEISAMNI
PYIIY 800

IAMTLRKPTHTKRLGFSTGIWTSLIIIFKMIYQMEFIKEENLSVFCEDVFGLNDTVHAPEWIGLHKTDKIFTYTR
DYILL 880

TILFAFRSIVELRQSLYRYEHGDLTPIRGVLFNNITRKDADIDMCNCLKYFANFFFYRFGLEMCRVTAVITIGLR
SDMIS 960

VIYAAFLLATLSLKRETIAQIWPYSTTCLAVSFAWQYILCVGIPKAFCHVYPWTNWDHNMIEWLFLPDFMIPPNP
VKLYA 1040

DFFLLLFMSLQLRVFRIEASEHDYIGGSNKSVLDEERGRRTLLKVNSEEKVEEIKNAAVPDFIGKSDTLLDHAKR
IVFVH 1120

LWWLTLSMVFIAGTSRVTLFAFGYVVGCFLFLWVGNSLFLRPMRVALALLNKLILYNACVIFIKISLQVVGCVYM
SQMYK 1200

NYCWVLQLFGIACLKTGVRPEKIVDPAILAECNVPHREAGIFYDGMCFMFLLIQQRIFGSEYFKHVVAEIRAQKF
IASRG 1280

AEIIAKITKKQVEEAERKEDEILSKVKKKMDRIRMKHERGAELPRPSTLIYQTQKQEGGDVLSEASDTREHSAHM
TELSG 1360

EPSRPTFFTPSDSVSVQFPSEDLRASTTRKRTKSISSDSKAMKRMDSMSPSGKQRIRFASRAIAGAHETVDFESG
DEWSS 1440

DEEDKLEPDLLEEVGVKGLGPLQLLNYAFKKGAIKEALKESNEIEAAHARMEREMEDAFGVADHNAIRRAILRQK
ARRPK 1520

ADYDESTFDEESITRREELPSLLRSTLGAVAAQPRGKKRPSPSALSRMPSIHDDSIREASEERSQRSGEGQEPAP
PTNVG 1600

RRRSSVHPLLEAPAIAKSPSSVADAIQALAVLEMCVVEKQNRDAAEEEQMRLLGAHRSRLQQLKLRLTELARNLG
TFALL 1680

FGRGVIESTIENLMSLSRDYRYIARVLTTEKELHKEMLIRVEDESDLRTALRNRRKSLVRHRCAENSKMVGIDES
FVQLH 1760

RYVQSTEDSAGQSTPASKAETASIVTTKPAVKKERDHMLIRLIQALYYVLLSRSEIICYFMIVLNQMHSASILSM
PLPLM 1840

TLLWGTLTVPRPSKNFWITMITYTQAMVIVKYIFQFGFFPWNKITASVHPFWPPRIIGIEKKDKYAAWDLALLMA
LFFHR 1920

NILKSIGLWDEKASAIVQEDTTTSRRRSSNCRATPTRLFSLTKTAIPVEADHDETSLSSRSSSSSSDGALKRKLS
MMKSA 2000

AEQLINKRKVIKKTNARLNAIRRFFSVLLNPVDRFPLDLYAPMFLCDVICFLIIIFGYSGFGADAGGVDGGVTAY
FEENK 2080

VPGTLVSMLILQFVLIIILDRAIFLRKFLFAKIVFQVVLVVFVHVWMFFLLPAATDRMFVTIFPCKLFYLTKIVYF
LISAK 2160

QIQAGYPKRSLGNIITNSYTMINWVLYKAFMLIPFLFELRALMDWMWMDTSLGVGDWFMLNDIYSHVSMIKCERN
IEEDY 2240

PSPKGVKKRPILKYGLGGVLLTAIILIIWFPLVIFSMANTVGTRSLPVECTCKLTIAGFEPLFKSTAQLSDIREL
TYEEY 2320

DNFQYTYRTSKQAQAYMADYTNRDVVQANINGNSSSRWSISPPSRTALVRELRGEQRMSLKFEWYFKRAPDENLQ
FGTAE 2400

DFRVINLEPRDPIRLDLADVIASGSRKLIRVPHLLIPMVKVPGEGKSDHVHALLSVHLKNDDDSIESTFYDGLLQ
LDSMD 2480

GIEWWKLRMVDPSFDPVIPKEEVILDNIVIYGFVDKVFPVTFSIITGGGILSLYLSMVLVFGRLMRSVVTGAMQR
IMFEE 2560

LPNVDRILRLCLDIYLVREAGELQLEEDLFAKLVFLFRSPATLIKWTKEKTT

....
..... 80

....
..... 160

....
..... 240

....
..... 320

....
..... 400

....
..... 480

```
...........................................................................
.....      560
...........................................................................
.....      640
.................................N.........................................
.....      720
...........................................N...............................
.....      800
........................................N...........N......................
.....      880
.................................N.........................................
.....      960
...........................................................................
.....     1040
............................N..............................................
.....     1120
...........................................................................
.....     1200
...........................................................................
.....     1280
...........................................................................
.....     1360
...........................................................................
.....     1440
...........................................................................
.....     1520
...........................................................................
.....     1600
...........................................................................
.....     1680
...........................................................................
.....     1760
...........................................................................
.....     1840
...........................................................................
.....     1920
...........................................................................
.....     2000
...........................................................................
.....     2080
...........................................................................
.....     2160
...........................................................................
.....     2240
...........................................................................
.....     2320
................................N..........................................
.....     2400
...........................................................................
.....     2480
...........................................................................
.....     2560
....................................................
2640
```

(Threshold=0.5)

SeqName	Position	Potential	Jury agreement	N-Glyc result
AgR007_g063_t01	674 NQSR	0.5608	(8/9)	+

```
AgR007_g063_t01  764 NYTW   0.7503     (9/9)   +++
AgR007_g063_t01  841 NLSV   0.6626     (8/9)   +
AgR007_g063_t01  853 NDTV   0.5926     (7/9)   +
AgR007_g063_t01  914 NITR   0.5797     (6/9)   +
AgR007_g063_t01 1069 NKSV   0.5641     (7/9)   +
AgR007_g063_t01 2353 NSSS   0.5316     (6/9)   +
----------------------------------------------------------------------
```

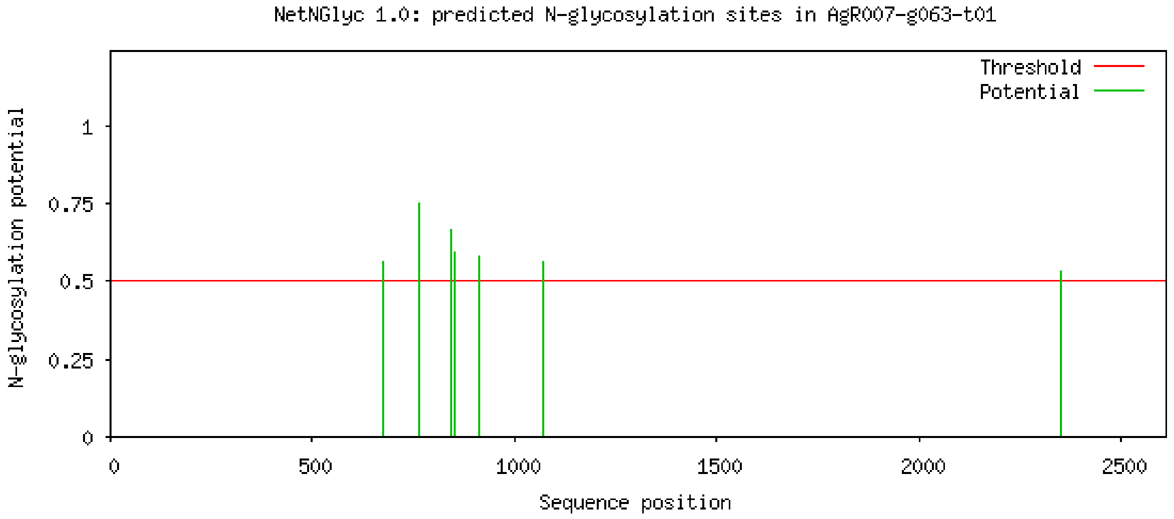

[Graphics in PostScript]

Output for 'AgB13X_g096_t06'

```
##########################################################################
#############

    Warning: This sequence may not contain a signal peptide!!

    Proteins without signal peptides are unlikely to be exposed to
    the N-glycosylation machinery and thus may not be glycosylated
    (in vivo) even though they contain potential motifs.

    SignalP-NN euk predictions are as follows:

  # name                 Cmax  pos ?  Ymax  pos ?  Smax  pos ?  Smean ?  D
?
  AgB13X_g096_t06             0.205  31   0.168  31   0.204  10   0.144   0.155
N  0.450      SignalP-noTM

    SignalP output is explained at
https://services.healthtech.dtu.dk/services/SignalP-4.1/output.php

##########################################################################
#############

Name:  AgB13X_g096_t06        Length:  2268
RFRVIRICNDLPREIAGVQILSVFMSCLESFHPSTIAYLVHRLPCSHGKVHYSIVASGASGFSIDYDNGMVFLRE
KLDTR      80
```

LSPVSLIIRAKDSSQPAQSSTVTCTVYVADVNDHAPLFVASQQQIFIDENTPLGHEVGRVFAVDQDVGTNGIVRY
SLDEG 160
SSSGFEINAISGSIKIRGELDREKNETHILKVRAKDGGDPPLSDTIIITIHLRDVNDNAPYFEPDVYNVTVPEST
PRGAQ 240
IISVTAKDDDKDQRITYKIQRMDRDIFTLTDLGEQGALLSLSDSFRNTDDVIEVIVSATDQGGLKGMCTVFIVVS
DVNAP 320
PSFLTHPFTVRISEHSAIGSEVIHLQAEDGDRGTNAMITYTIDSPDFTIDEKTGLITVASDLDREVRTTYVVNVT
VQDHA 400
SPPLTASTTIEIILEDVNDNAPVFSSSNYTMTISEDTPVGTSFMQITATDLDAGSNAFVDYFIDVNDKRALKVDA
FKLDR 480
SSGTLRVHKKLDREQNDIHVIPVIARDRGNPSLSSSATVTVVLSDVNDNAPQFESCRYDLWIAENSPIGTVVGTI
VARDR 560
DVGHNARIHFKIFGGVDAKLFDIEADPNQEGVVHILSRKIFDYEAKNNKFYLEIQASSGQLSSTVPIYVHVSDVN
DNKPQ 640
LRDFVVAIASYDGESFDPQIGTVPAFDPDHNATLEFHVESNDVILVDRFSGALRTVSAWRRHIEAHYKACVSDGP
NTACA 720
MCHVIYTPVDESSLRESVTARIDDMNQDEFLDYHTFRRFITAISLLDRWYPKDVHVFSVHSEGRLTNVTFFVRHK
GRLQR 800
SRRIQELIRDAKGRLNDVSGMNIDVLWDESCASEPCPYYQQCRQVHKYLHSAQRFKTDSFLMRSLDVVGTFTCEC
SKGFA 880
GSDALQQLCNERIDQCYSSPCHHQGTCIPLENGYRCDCPPGRIGTNCEGTIFSDACLPHSCFSEALCAIKNRTIF
CENCK 960
WAKHDTDELCRLRSLAFTGEGYVALPIAVPRMEFKIEFSLATTSGSGVVLFAGDLKSDFLEVSLEDALLGARFSL
GKEIF 1040
EGRMEDWRVNKVNDGNWHKVTVDFYDHKLVLSLDACEPYISMAFSNTTGYGKCATEVVANLPEKCADGSVTCHRF
LDVVP 1120
VVYFGARPGVASKSEYNTVLNDESLMPVVQHGYTGCISNLSVDGLLYHFSSFSELSRSGVVLAGCKEKREVCSSM
PCHPS 1200
SRCENYWGGHHCRCEHQLHTEHACSAERPSYVMLDDEESYVMWKVHNADVFRNVSLEFRTRSRDTQVIAVEFVLH
SDFIT 1280
FSLAQGYGVVTVGRQQFTLTFPYFSDGKFKAILIGIDGMFVRVLVDYIHEKRFPLSEGEAVLGVRSMYSGLAPSM
SHPQR 1360
FEGCLRNVKLNNLRAKVVEQSRTKSNCQVRNECGQPHICPRNSRCIRDWDRHRCHCLKGYIGDNCTDACAFPRIC
AHQGF 1440
CSRSNNSYGYECRCADGFTGRNCERKAVTRTCPLGWYGKFPHCRECECDVRRGFIRQCDEKTGTCLCEPGNYFSI
DRCVP 1520
CECGFGSSGKSCSALGQCQCTGEALGRRCDRCAHAAEELDRQTLKCVKVHNRCPSNIEQGIQWPTTVHGVVARQS
CSFGE 1600
TGIALRKCGNNGKWEQVNSYNCTFFAYQKMADAASNIELAQMLANATNDRWTMRGINLEIANDAVKRLLDAERKT
NATFH 1680
VRTEDFTRNILQSLNNLASVANNANYVRTSRAVLDIGAHLRVVHERNHYLNSFFFSGDKLVFSIDTLSQSDHHIL
PRFAN 1760
FVDDRTESLRSVTIQLLCSLRQTDVIFYAIFHEPKCHGCENSIVAVLLDDRPCTIRISFPVVESNGWTYPECVRL
GAVQA 1840
NNATSSDLPHPESKDILFSRWSSEGSTLAALNSTHVVCQFQQFGVFTIFMRTDRGALIRFTLPHSIPYTGPLSAA
FALLL 1920
TLLSASATICRGSIRTRIVRFGFIISFLLDASTIFFIHRIPPSNVFCPVQNAVVSFCTSALFAWLFLYALRIYCF
FLNGY 2000
TQPNLTMVLVIGIIVPVLLSSLTFFFAPGCSLRVYAWFFWILVTPIILLLLLNLYALLTSLIISTKKQFDIVVSR
FSPRK 2080
TLILHLILTAFCVAYNTFGLFLLYDRQTNPLLEAATNVILVLFSAYLFLWSGYFGRESVSAHSGELWVNDASKAA
IGSSE 2160
RQCNSPLLPLTDALDTYAAIKMNGMRQWMPDIIPSATCVHQERPITTLPRPNILSPATKVLHHEPVYGNVSIIRP
ISSKF 2240
YHTSDDDMEHAYGSYTSKRFYPSSTFTR

....
..... 80
....
..... 160
........................N..N.......
..... 240

```
...........................................................................
.....     320
........................................................................N..
.....     400
...........................N...............................................
.....     480
.............................N.............................................
.....     560
...........................................................................
.....     640
..............................N............................................
.....     720
..................................................................N........
.....     800
...........................................................................
.....     880
......................................................................N....
.....     960
...........................................................................
.....    1040
...........................................................................
.....    1120
......................................N....................................
.....    1200
....................................................N......................
.....    1280
...........................................................................
.....    1360
...............................................................N...........
.....    1440
...........................................................................
.....    1520
...........................................................................
.....    1600
............................................N..............................
.....    1680
...........................................................................
.....    1760
...........................................................................
.....    1840
.N.............................N...........................................
.....    1920
...........................................................................
.....    2000
...N.......................................................................
.....    2080
...........................................................................
.....    2160
....................................................................N......
.....    2240
............................
2320
```

(Threshold=0.5)

SeqName	Position		Potential	Jury agreement	N-Glyc result
AgB13X_g096_t06	185	NETH	0.5267	(6/9)	+
AgB13X_g096_t06	228	NVTV	0.7706	(9/9)	+++
AgB13X_g096_t06	393	NVTV	0.7421	(9/9)	++

```
AgB13X_g096_t06  428 NYTM   0.6909     (8/9)   +
AgB13X_g096_t06  510 NPSL   0.5933     (8/9)   +    WARNING: PRO-X1.
AgB13X_g096_t06  671 NATL   0.6216     (8/9)   +
AgB13X_g096_t06  787 NVTF   0.6971     (9/9)   ++
AgB13X_g096_t06  951 NRTI   0.5667     (6/9)   +
AgB13X_g096_t06 1086 NTTG   0.4741     (6/9)   -
AgB13X_g096_t06 1159 NLSV   0.5124     (5/9)   +
AgB13X_g096_t06 1253 NVSL   0.5330     (6/9)   +
AgB13X_g096_t06 1424 NCTD   0.7162     (9/9)   ++
AgB13X_g096_t06 1445 NNSY   0.3926     (7/9)   -
AgB13X_g096_t06 1621 NCTF   0.4269     (7/9)   -
AgB13X_g096_t06 1645 NATN   0.5370     (6/9)   +
AgB13X_g096_t06 1676 NATF   0.4774     (6/9)   -
AgB13X_g096_t06 1842 NATS   0.5590     (6/9)   +
AgB13X_g096_t06 1872 NSTH   0.5477     (5/9)   +
AgB13X_g096_t06 2004 NLTM   0.5612     (6/9)   +
AgB13X_g096_t06 2229 NVSI   0.6876     (9/9)   ++
----------------------------------------------------------------------
```

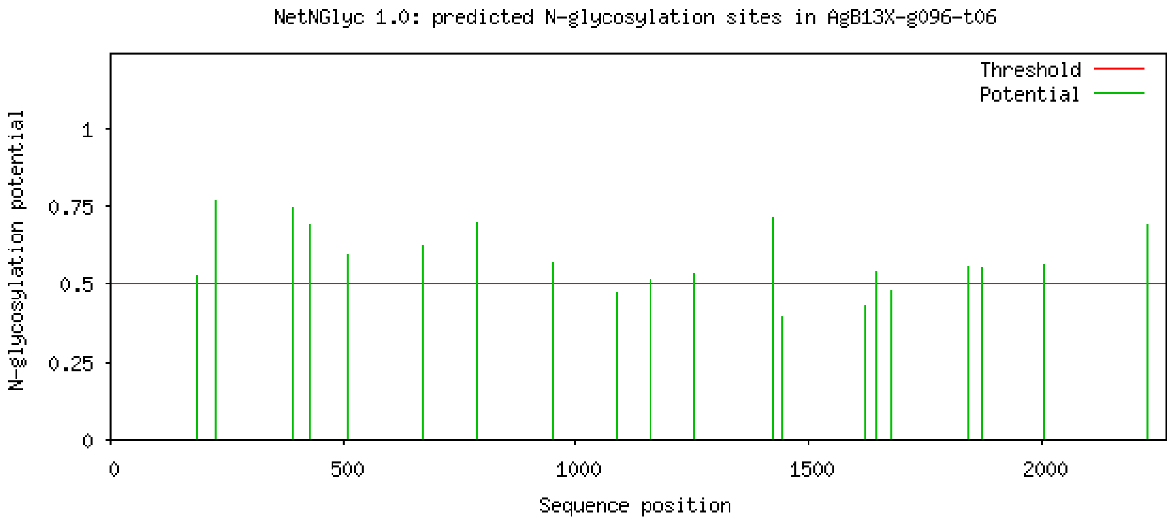

[Graphics in PostScript]

Output for 'AgR007_g282_t05'

```
##############################################################################
#############

    Warning: This sequence may not contain a signal peptide!!

    Proteins without signal peptides are unlikely to be exposed to
    the N-glycosylation machinery and thus may not be glycosylated
    (in vivo) even though they contain potential motifs.

    SignalP-NN euk predictions are as follows:

  # name                 Cmax  pos ?  Ymax  pos ?  Smax  pos ?  Smean ?  D
?
```

  AgR007_g282_t05   0.115 45 0.135 20 0.214 12 0.156   0.146
N 0.450   SignalP-noTM

    SignalP output is explained at
https://services.healthtech.dtu.dk/services/SignalP-4.1/output.php

##
#############

Name: AgR007_g282_t05   Length: 1889
MSVLASMMASSAEEDDHHAEDAQKSDLWQQTLQAAVAASSQSDAAKKRQQQRKPLRQANVVERSERSLLCLTLSN
PLRKA   80
CISIVEWRPFEWLILFMICANCIALAVYQPYPAQDSDTKNTILEQIEYLFIIVFTIECILKVIALGFLFHPGAYL
RNAWN   160
ILDFIIVVIGLVSTALSRMNIQGFDVKALRAFRVLRPLRLVSGVPSLQVVLNAILRAMIPLLHIALLVMFVIIIY
AIIGL   240
ELFCGKLHSTCVDPATGQLAQHTPSPCGFATTAFHCQPSGHYEGVKWICTSNTTWQGPNNGITNFDNFGLAMLTV
FQCVS   320
LEGWTDVMYWVNDSVGHEWPWIYFVTLVILGSFFVLNLVLGVLSGEFSKEREKARARGLFQKFREKQQLEEDLKG
YLDWI   400
NQAEDIEPVNDDEQEDEQQFTGEEMDEEGEDKTDDSKPSWFSKRLRRLQKFNRRCRRGCRRLVKSQTFYWLVIIL
VLLNT   480
LVLTSEHYKQEPWLDHFQTIANLFFVVLFSLEMMLKMYSLGLTTYTTSQFNRFDCFVVISSIIEFVCVYFDLMKP
LGVSV   560
LRSARLLRIFKVTKYWTSLRNLVSSLLNSLRSIMSLLLLLFLFIVIFALLGMQVFGGKFNFNPMNPKPRANFDTF
IQALL   640
TVFQILTGEDWNTVMYNGIASFGGVGSWGVLVSVYYIVLFICGNYILLNVFLAIAVDNLADADSLTNAEKEEEQA
EVEEE   720
AAEDDYEDEKYDENCNEEARDDSRIVVDEEEDIGEIVTARPRRMSELAPMKQQKPIPKASSLFILSHTNPFRVFC
NKIVN   800
HSYFTNSVLVCILVSSAMLAAEDPLEAQSPRNTILNYFDYFFTTVFTVEITLKVVVYGLVFHKGSFCRNAFNLLD
ILVVA   880
VSLVSFVLKSDAISVVKILRVLRVLRPLRAINRAKGLKHVVQCVIVAVKTIGNIMLVTFMLQFMFAIIGVQLFKG
TFFKC   960
NDESKMTEQECRGEFLAYEDGDPMKPQRMRRDWTKNDFNFDNVADAMVSLFVVSTFEGWPDLLYVAINSNEEDRG
PVYNA   1040
RQAVALFFIAFIVVIAFFMMNIFVGFVIVTFQNEGEREYENCELDKNQRKCIEFALKAKPHRRYIPRNRFQYRVW
WFVTS   1120
QFFEYVIFIIILCNTTTLAMKHYPPDPAMDHVLDVLNLVFTGVFAFEALFKIIALNPKNYFGDRWNAFDFIIVLG
SFIDI   1200
IYGKLSPGSNIISINFFRLFRVMRLVKLLSRGEGIRTLLWTFMKSFQALPYVALLIVLLFFIYAVIGMQVFGKVA
LNDET   1280
HIHRNNNFHTFPAAILVLFRSATGEAWQEIMLSCSDREEVKCDPASDDYKQNPDARCGVDFAYPYFISFFMLCSF
LVINL   1360
FVAVIMDNFDYLTRDWSILGPHHLEEFVRLWSEYDPDAKGRIKHLDVVTLLRKISPPLGFGKLCPHRLACKRLVS
MNMPL   1440
NSDGTVCFNATLFALVRTNLKIYTEGNIDEVNEQLRSAIRRIWKRTPQKMLDEVVPPAGRDDDVTVGKFYATFLI
QDYFR   1520
RFKKRKELEAKGIIPQQTSQAMALQAGLRTLHEIGPELKRAISGNLETDFTFDGEEPQHRRPHSLFNNIMTALGG
TARSN   1600
HQEMPREERTSRLLPITANHPAVSPTHSLRGNDLMSLVAHSGVATNCSVNLPPSARSNGGLVQRRLPKIPPINLS
FDQKS   1680
YADDINTPRVAPYDHRYIVANRNIPLDVDEDEEWYERREANHVRHDERWTNDGMPERGLREPFLLARSQALAIAG
VPPDM   1760
SDAFEGTYRPAPDGKSVRLPFSSRPVLRPAESNEEGLTERLVGEALGLGRYMDERVVEAARREIAEAYSLEESEL
ESAAV   1840
ALTDARYLEHLGMERSEVRDFNRYSASALLRPAPSQETHEDDLLLVTTL
....
.....   80
....
.....   160

```
...........................................................................
.....      240
...........................................................................
.....      320
...........N...............................................................
.....      400
...........................................................................
.....      480
...........................................................................
.....      560
...........................................................................
.....      640
...........................................................................
.....      720
...........................................................................
....N      800
...........................................................................
.....      880
...........................................................................
.....      960
...........................................................................
.....     1040
...........................................................................
.....     1120
...........................................................................
.....     1200
...........................................................................
.....     1280
...........................................................................
.....     1360
...........................................................................
.....     1440
........N..................................................................
.....     1520
...........................................................................
.....     1600
...........................................................................
.....     1680
...........................................................................
.....     1760
...........................................................................
.....     1840
.................................................
1920
```

(Threshold=0.5)

SeqName	Position		Potential	Jury agreement	N-Glyc result
AgR007_g282_t05	292	NTTW	0.4570	(6/9)	-
AgR007_g282_t05	332	NDSV	0.5040	(5/9)	+
AgR007_g282_t05	800	NHSY	0.6111	(6/9)	+
AgR007_g282_t05	1134	NTTT	0.3930	(7/9)	-
AgR007_g282_t05	1449	NATL	0.6717	(9/9)	++
AgR007_g282_t05	1646	NCSV	0.4980	(5/9)	-
AgR007_g282_t05	1673	NLSF	0.3596	(9/9)	--

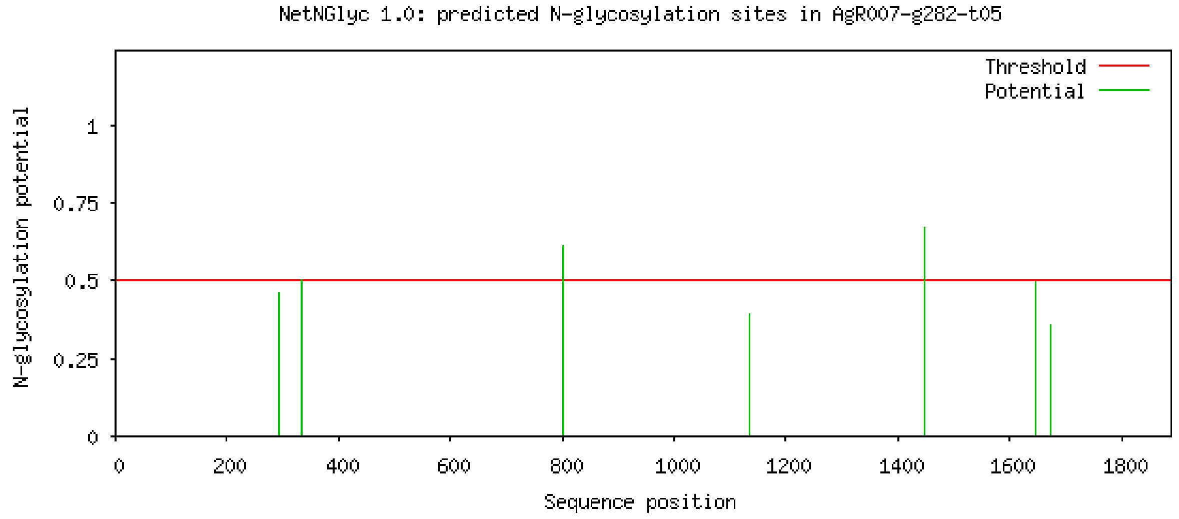

[Graphics in PostScript]

Output for 'AgB13X_g094_t05'

```
################################################################################
##############

    Warning: This sequence may not contain a signal peptide!!

    Proteins without signal peptides are unlikely to be exposed to
    the N-glycosylation machinery and thus may not be glycosylated
    (in vivo) even though they contain potential motifs.

    SignalP-NN euk predictions are as follows:

  # name                  Cmax  pos ?  Ymax  pos ?  Smax  pos ?  Smean ?  D
?
  AgB13X_g094_t05            0.110  29  0.111  12  0.131   7  0.111   0.111
N  0.450      SignalP-noTM

    SignalP output is explained at
https://services.healthtech.dtu.dk/services/SignalP-4.1/output.php

################################################################################
##############

Name:  AgB13X_g094_t05        Length:  1862
MLQQPVARELRSFQSLSRFSGPRAILNRRQSTLNARRLSQSRRKNTEAIVDDSQASSATGACVVPAEYVRAQPPR
SKAVS      80
LSLEASPMHPWYGSGEPSEQQRQPQVEWHDEADFIDDECDLPYPGFVEPALHCLKQASPPRLWALRMVMNPWFDR
LTMIV     160
ILINCITLGMYRPCEDGGECTTYRCYVLSLIDHTIFAYFALEMVIKIIALGFYGPAAYLSDTWNRLDFFIVCAGC
AEYLL     240
QEYLGNINLTAIRTIRVLRPLRAVNRIPSMRILVNLLLDTLPMLGNVLLLCFFVFFIFGIVGVQLWAGLLRNRCV
INLPK     320
TNMAINVTDVSLTRYYIPEDTSLEYICSQSDSSGLHTCNKLPPYTHNGVKCNLTLFEWEKVSNESCINWNIYYNE
CTVMH     400
```

RNPFQGSVSFDNIGFAWVAIFLVISLEGWTDIMYYVQDAHSFWNWIYFVLLIVVGAFFMINLCLVVIATQFAETK
RRETE 480
RMIQERKRLRSSGSLSGSEQGVTSSKDGAGGDSVYAAIVRFISQQSRRLKRRLLKEIRVLRLRFFSKQNSTAANN
STAAL 560
SAVEAGNRPETSDIQKISQERAEENMSAEGKRRGSRSMKRKRRRSSLISKKTQSNGSVQKVYGGTSADSFARPPL
IPHGS 640
SLSKDSSSGEESDVEDSDKWSASETRAEDLDVRQNHRGSTLNEDRIQGASPIPTAPKNSRIGWLRGRIQAFVVCN
HFKRG 720
ILFAILINTLSMGVEYHQQPEILTVILEYSNYFFTGLFALEMLLKIIADGLFGYLSDGFNLFDGGIVALSVLELF
QEGKG 800
GLSVLRTFRLLRILKLVRFMPALRYQLVVMLRTMDNVTVFFGLLVLFIFIFSSLGMVLFGCKFCTDSSNGERCIR
RQMAD 880
ASQTSCECERTNFDSFLHATVTVFQILTQEDWNMVLFNGMAQTTPWAALYFVALMTFGNYVLFNLLVAILVEGFQ
ESKEE 960
EKRQLEEEARKKANEEEEERKRELELLLAKTSSPSFILSRKTDTKQCTCGNGHMAQLGAYDARIPLLPFAPDDIA
SLHEI 1040
SVGSQVVHGASAGAAEENKICGDHLYDQRTTAQRSKLGTSDERCSQYSAVRSIRFNAHDEGSDSETDDENKALRN
KHRLN 1120
RHTSLVLPRLSTSYDIYGRGARQRMNSWCGLHGIFSPCCPVHGRQALIEAYARDKFIQASQELQKAIAEEERREE
QRQNS 1200
MWRRLYRKTCMHKRADHSLYIFSPKNGVRIRCLQLTQKKWFDYTILFFIGINCITLAMERPSIPPKSVERQFLTI
SGYVF 1280
TVIFTIEMTLKVIANGCLIGPGAYFKDGWNILDGTLVIISLVNVGFELLVHGDSPKIFGVIRVLRLLRALRPLRV
INRAP 1360
GVKLVVMTLISSLKPIGNIVLICCTFFIIFGILGVQLFKGMMYHCIGHDISNVTTKAECLEDPRNKWVNHRYNFD
NLGQA 1440
LMSLFVLSSKDGWVSIMYQGIDATGVDMQPVENYNEWRMIYFISFLLLVGFFVLNMFVGVVVENFHKCKEALEAE
MRERA 1520
RQKRLERKLKRQQFEDQFGHKRKKKEKSHPYWYNYGETRMFLNGIATSKYFDLAIAAVIGINVISMAMEFYMMPA
GLKYV 1600
LKALNYFFTAVFTLEAAMKLIALGIKRFFKERWNQLDMFIVVLSIAGIIFEEFEALELPINPTIIRVMRVLRIAR
VLKLL 1680
KMAKGIRSLLDTVGEALPQVGNLGSLFFLLFFIFAALGVELFGKLECSEDHPCDGLGEHAHFKNFGMAFLTLFRI
ATGDN 1760
WNGIMKDALRDDCDPSEHCESNCCVDPILAPCFFIIFVLISQFVLVNVVVAVLMKHLEESNKREESADDIAAGNA
AVGTD 1840
TDVNKTDTDIEDNLDSSSRKDP
....
..... 80
....
..... 160
....
..... 240
.......N...
..... 320
.....N...N..........N............
..... 400
....
..... 480
....N.....N
..... 560
........................N..............................N...................
..... 640
....
..... 720
....
..... 800
...................................N.......................................
..... 880
....
..... 960

```
...........................................................................
.....    1040
...........................................................................
.....    1120
...........................................................................
.....    1200
...........................................................................
.....    1280
...........................................................................
.....    1360
...................................................N.......................
.....    1440
...........................................................................
.....    1520
...........................................................................
.....    1600
............................................................N..............
.....    1680
...........................................................................
.....    1760
...........................................................................
.....    1840
...N..................
1920
```

(Threshold=0.5)

SeqName	Position		Potential	Jury agreement	N-Glyc result	
AgB13X_g094_t05	248	NLTA	0.7928	(9/9)	+++	
AgB13X_g094_t05	326	NVTD	0.7730	(9/9)	+++	
AgB13X_g094_t05	372	NLTL	0.7101	(9/9)	++	
AgB13X_g094_t05	383	NESC	0.5619	(5/9)	+	
AgB13X_g094_t05	549	NSTA	0.5848	(7/9)	+	
AgB13X_g094_t05	554	NNST	0.3543	(8/9)	-	
AgB13X_g094_t05	555	NSTA	0.5411	(7/9)	+	
AgB13X_g094_t05	585	NMSA	0.5166	(6/9)	+	
AgB13X_g094_t05	615	NGSV	0.6143	(8/9)	+	
AgB13X_g094_t05	836	NVTV	0.7668	(9/9)	+++	
AgB13X_g094_t05	1412	NVTT	0.5226	(6/9)	+	
AgB13X_g094_t05	1661	NPTI	0.6460	(9/9)	++	WARNING: PRO-X1.
AgB13X_g094_t05	1844	NKTD	0.5902	(7/9)	+	

NetNGlyc 1.0: predicted N-glycosylation sites in AgB13X-g094-t05

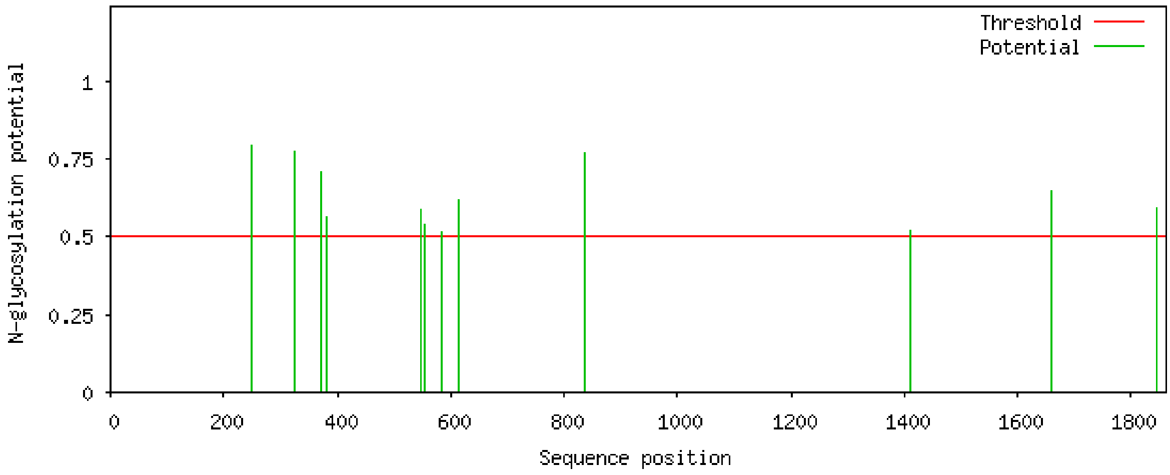

Supplement: Supplementary Table 4 — Glycosylation assessment. [file Table_4.pdf]
